# Ankle Blood Pressure and Pulse Pressure as Predictors of Cerebrovascular Morbidity and Mortality in a Prospective Follow-Up Study

**DOI:** 10.4061/2010/729391

**Published:** 2011-01-17

**Authors:** Heikki J. Hietanen, Rauni Pääkkönen, Veikko Salomaa

**Affiliations:** ^1^Department of Clinical Physiology, Helsinki Deaconess Institute, Alppikatu 2, 00530 Helsinki, Finland; ^2^National Institute for Health and Welfare (THL), Helsinki, Finland

## Abstract

*Background and Objective*. We examined the association of elevated ankle blood pressure (ABP), together with exercise blood pressure, with incident cerebrovascular (CV) morbidity and mortality in a prospective follow-up study of 3,808 patients. The results were compared with pulse pressure, another indicator of arterial stiffness. *Methods*. Patients with normal ankle and exercise brachial blood pressures were taken as the reference group. Pulse pressure was considered as quartiles with the lowest quartile as the reference category. *Results*. A total of 170 subjects had a CV event during the follow-up. Multivariate adjusted hazard ratio of a CV event was 2.24 (95% CI 1.43–3.52, *P* < .0001) in patients with abnormal ABP. The pulse pressure was significant only in the model adjusted for age and sex. *Conclusion*. The risk of a future CV event was elevated already in those patients among whom elevated ABP was the only abnormal finding. As a risk marker, ABP is superior to the pulse pressure.

## 1. Introduction

High ankle blood pressure (ABP) might be an emerging risk marker for early subclinical damage of arterial vessels evaluated as elevated ankle blood pressure alone [1, 2] or in conjunction with the elevated ankle brachial index (ABI) [3–5]. The high ABP or high ABI provides an indication of arterial stiffness or atherosclerosis of the vessel wall whereas the low ankle pressure or low ABI is indicative of advanced atherosclerosis, which has reached the point where the blood flow is impeded. Several studies have demonstrated that there is a nonlinear, U-shaped association between the ankle brachial index and total mortality or cardiovascular events [4–10]. On the other hand, while the normal ABI does not exclude significant peripheral arterial disease [6, 11, 12] the ABP alone could be useful in evaluating early vascular changes. 

 The pulse pressure (PP) has been used as a crude indicator of arterial stiffness [13–17]. The increased PP predicts dementia [18] and cardiovascular mortality [15], acting more on coronary than cerebrovascular (CV) vessels [19]. In hypertensive subjects with high PP, stroke mortality is increased [20, 21]. In many recently published papers the central PP is more closely related to vascular damage than the peripheral PP [13, 22–26], and the peripheral pressure does not necessarily accurately reflect the central pressure. The difference between central and peripheral PP is dependent on age, height, and heart rate making the evaluation of aortic pulse pressure from a peripheral pulse pressure difficult.

 Elevated ABP may provide a pivotal opportunity to identify persons at high risk of stroke or TIA. Accordingly, the main aim of the present study was to assess the utility of ABP, together with exercise brachial BP, as a predictor of CV events in a prospective setting. The second aim was to investigate whether the elevated ABP predicts CV events better than the pulse pressure. Both measurements reflect aortic stiffness. We hypothesized that the ABP would be superior to the pulse pressure for screening purposes.

## 2. Material and Methods

This prospective follow-up study was initiated in August 1989. The methods of baseline data collection have been described in our previous paper [1]. The subjects were derived from a group of 4,038 consecutive ambulatory patients who underwent a symptom-limited bicycle exercise test at the Deaconess Institute between August 1989 and December 1995. Patients with a history of cardiovascular disease (including those with a history of cerebrovascular events) at baseline investigation were excluded from the analyses, and the final study group consisted of 3,808 patients. The study was approved by the Ethical Committee of the National Public Health Institute of Finland. 

**Figure 1:**
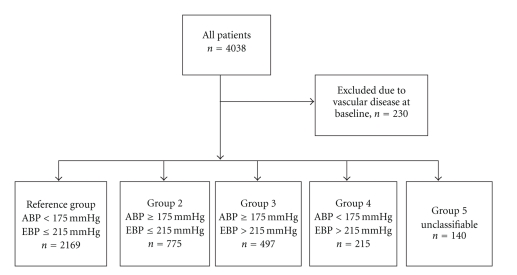
A flow-chart describing derivation of the blood pressure groups from all consecutive patients undergoing a clinical exercise test during the period 1989 to 1995. ABP: ankle blood pressure, EBP: brachial exercise blood pressure at the level of 150 Watts for men and 80 Watts for women.

The ankle and brachial blood pressures were obtained simultaneously after a 5 min rest in supine position using the Doppler sonography for the ABP and the standard mercury sphygmomanometer for the left arm blood pressures. Subjects were divided to five groups based on the ABP at rest and exercise blood pressures (EBPs) at a moderate exercise level (men 150 Watts, women 80 Watts) as shown in the flow chart (Figure 1).

 The reference group consisted of “normal” patients with normal ABP (<175 mmHg) and normal systolic EBP (≤215 mmHg). Group 2 consisted of patients with elevated ABP (≥175 mmHg), but normal EBP. Group 3 had elevated ABP and exaggerated EBP (>215 mmHg). Patients in group 4 had elevated EBP, but the ABP was normal. The discrepancy between the ABP and EBP indicates significant atherosclerotic changes in conduit vessels. Group 5, the unclassifiable group, consisted of patients, who could not tolerate the moderate exercise level. 

The follow-up was 16 years, until the end of 2007 using record linkage of the study data with the National Hospital Discharge Register and the National Causes of Death Register. The endpoint was a major CV event, that is, death due to CV, nonfatal stoke, or a transient ischemic attack (TIA). The 9th (until 31.12.1995) and 10th versions of the International Classification of Diseases (ICD-9 and ICD-10) were used for coding of causes of death and hospitalizations. We took as CV events the ICD-9 codes 431, 436, 4330A, 4331A, 4339A, 4340A, 4341A, 4349A, and 435 or the ICD-10 codes I61, I63-I64. G45. These registers cover all deaths and hospitalizations in Finland. Thus, the coverage of follow-up was 100% for symptomatic stroke events leading to hospitalization or death in Finland. It is however likely that all TIAs have not been hospitalized and those treated on an ambulatory basis are not identified as CV events in the present study. Altogether, 170 CV events were observed during the follow-up. Of them, 31 were fatal. As a whole, the study consisted of 53,044 person-years of followup.

### 2.1. Statistics

Data are expressed as mean ± SD for continuous variables or counts and proportions for categorical variables. The following cardiovascular risk factors were dichotomized: early parental cardiovascular death (yes or no), self-reported elevated cholesterol (>6 mmol/l, yes or no), self-reported elevated blood glucose (>6 mmol/l, yes or no), and current smoking (yes or no). Age, BMI, smoking (years, packet/day), and blood pressure (mmHg) were handled as continuous variables. Student's *t*-tests were used for comparisons of normally distributed variables, between persons with and without a CV event during the follow-up. Categorical variables were compared using chi-square tests. 

 Associations between the blood pressure groups, and CV mortality and morbidity (first events) were analyzed using Kaplan-Meier curves and log-rank tests. Cox proportional hazards models were used for estimating the multivariate-adjusted independent associations of the blood pressure groups or the quartiles of pulse pressure with the risk of a CV event. Results were expressed as hazard ratios (HRs) and 95% confidence intervals (CIs) compared to the “normal” group or to the lowest quartile of pulse pressure. The basic models were adjusted for age and sex. The larger models were further adjusted for BMI, physical working capacity (METs), self reported blood glucose and cholesterol, current smoking, resting brachial systolic blood pressure, and early parental history of cardiovascular disease. The statistical analyses were carried out with R (Version 2.11.1).

## 3. Results

Baseline characteristics of the study participants are outlined in Table 1, stratified by the CV event. A total of 170 subjects developed a CV event (30 TIAs, 109 nonfatal strokes and 31 fatal strokes) during the follow-up. The patients with a CV event were older and the blood pressures were higher. In patients without a CV event almost all other risk factors were more favorable compared with the CV event group.

**Figure 2:**
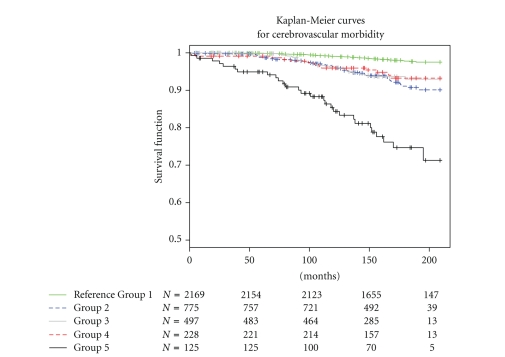
Kaplan-Meier curves for cerebrovascular (CV) events according to specified blood pressure group. Log-rank test for the difference between the blood pressure groups: *P* < .0001. The numbers indicate people remaining in the follow-up at different points in time. The reference group consists of patients with normal brachial pressure (<140 mmHg), ankle blood pressure <175 mmHg and exercise blood pressure ≤215 mmHg at the moderate exercise level. Group 2 had elevated ankle blood pressure (≥175 mmHg), but normal exercise blood pressure. Group 3 had elevated ankle and exercise blood pressure. Group 4 had ankle blood pressure <175 mmHg but elevated exercise blood pressure (discrepancy), and group 5 could not be classified because of poor exercise tolerance.

Figure 2 shows Kaplan-Meier curves for CV event in different blood pressure groups. The curves diverge continuously and significantly (*P* = .001, log rank test) throughout the 16 years of follow-up.

The patients with elevated ankle blood pressure with or without exaggerated exercise blood pressure had 2.6-2.7-fold risk of a CV event compared with the reference group in the age- and gender-adjusted model (Table 2). In the wider model the hazard ratio was 2.2–2.4. In patients with obstructive changes in leg arteries (Group 4) the risk of a future CV event was 2.7-fold in the basic model and 2.4-fold in the multivariate adjusted model. The greatest hazard ratio (7.8 in the basic model and 5.8 in the wider model) was found in the unclassifiable patients (Group 5). In this group, 55 patients had ABI < 1.0, and 25 patients had ABI < 0.9.

Pulse pressures in the five blood pressure groups were 42.7 ± 11, 54.4 ± 15, 58.5 ± 14, 49.0 ± 14, and 52.6 ± 16 mmHg, respectively. Correlation between the pulse pressure and the ankle blood pressure in the blood pressure groups I–III was high (*r* ^2^ = 0.48). The correlation was lost, however, when obstructive changes were observed in the conduit vessels (Group 4) (*r* ^2^ = 0.11). The risk of a CV event by quartile of pulse pressure is shown in Table 3. The reference category is the lowest quartile of the pulse pressure. Significantly elevated risk for a CV event was found only for the fourth quartile in the smaller model.

## 4. Discussion

The main finding of our study was that ABP, when considered together with EBP in a physiologically meaningful way, improved the prediction of incident CV events independently of classic risk factors. The healthy group with normal ABP and EBP had the best prognosis, while the group with poor exercise tolerance had clearly the worst prognosis. For groups II–IV, the Kaplan-Meier curves were overlapping. Furthermore, ABP was a better predictor than the pulse pressure. Increased aortic and conduit vessel stiffness with high pressure pulsatility may be the explanation for our finding. The elevated ABP may be a useful surrogate marker for central and conduit arterial stiffness.

These findings extend our understanding of the behaviour and clinical significance of the ABP. In the recent PARTNER study [4], individuals with high ABI had much higher systolic blood pressure in the ankle and modestly lower brachial artery systolic blood pressure compared with those with normal ABI. The pathophysiologic conditions causing stiffer arteries or stiffer surrounding tissues are insufficiently understood. While diabetes and degenerative vascular changes explain the phenomenon only in part [6], the increased stiffness, central and peripherial, could partly explain the elevated ABP. Aortic wall stiffness increases throughout the normal human lifespan, particularly in the presence of cardiovascular disease risk factors, and is first seen in increasing pulse wave velocity and central wave reflection (augmentation) [16, 27–29]. The stiffening of the aorta with minimal stiffening in the large peripheral muscular arteries decreases the normal impedance mismatch between the central and peripheral arteries enabling the transfer of excessive pulsatile energy into the periphery [17, 27]. Further research is needed to unravel the relationship of ABP to the pulse wave velocity which is a standard measure of the central aortic stiffness.

 When the conduit vessels are free of flow limiting atherosclerotic stenoses, the main determinants of ABP are systolic blood pressure, enhanced pulse pressure, and local changes caused by rigidity of the arteries in the lower extremities. In these conditions, we can draw epidemiologic conclusions from ABP per se without indexing it to the brachial systolic pressure. The discrepancy between the ABP and exercise blood pressure may disclose those patients among whom stenotic changes along the conduit vessels decrease the ABP. 

The good correlation between PP and ABP is lost when the stenotic changes limit the flow in the conduit vessels. The PP as a crude index of central arterial stiffness is not only hampered by age and heart rate but also by flow limiting atherosclerotic changes in the conduit vessels. That may explain why the peripheral PP is differently related to the risk of CV events in the literature [14–16, 19, 20, 23–26, 30] and why the peripheral PP has predictive value for ischaemic stroke also in normotensive patients [19, 31] among whom the flow limiting atherosclerotic changes are minor. 

A strength of our study is the large sample size and the long follow-up period of 16 years. Also, in Finland the record linkage to the National Hospital Discharge Register and the National Causes of Death Register has good accuracy and coverage [32]. Some limitations should be acknowledged, however. The persons studied do not represent a random sample of the general population. The study participants are better educated and have a higher than average socioeconomic position. The ABP was measured from one leg only. The blood glucose and total cholesterol were self-reported and only half of the persons knew their glucose value. The persons without a known abnormal cholesterol or glucose values were taken as normal. The study was also limited by the lack of stroke subtyping. However, as shown by the ICD codes listed in Section 2, we excluded subarachnoid hemorrhages and included only intracerebral hemorrhages and ischemic strokes to keep our material more homogenous. Furthermore, since ischemic strokes generally account for approximately 75%–80% of all strokes, the overwhelming majority of included strokes were of ischemic origin.

In conclusion, our results suggest new ideas about surrogate markers of subclinical vascular damage. The elevated ABP reveals better than PP the early adverse vascular changes, and the discrepancy between ABP and EBP discloses those patients in whom the atherosclerotic changes have propagated to the arterial lumen causing flow limiting stenosis. The ABP measurement may help us to better identify the patients who have the greatest risk for a CV event. 

## Figures and Tables

**Table 1:** Baseline characteristics of participants with and without incident cerebrovascular event during the follow-up.

	No cerebrovascular event	Cerebrovascular event	*ρ*
	*n* = 3639	*n* = 170	
Age (years)	50 (10)	57 (10)	0.0001*
Men/women, *n* (%)	2423 (67)/1216 (33)	105 (62)/65 (38)	NS
Body mass index (kg/m^2^)	26.1 (4)	26.6 (4)	NS
Syst. blood pressure (mmHg)	133 (18)	142 (19)	0.0001*
Diast. blood pressure (mmHg)	85 (11)	89 (10)	0.0001*
Pulse/min	74 (13)	74 (12)	NS
SBP80 (women)	181 (25)	193 (23)	0.001*
SBP150 (men)	201 (27)	212 (30)	0.0001*
SBP Max	203 (28)	205 (29)	NS
Ankle blood pressure (mmHg)	165 (27)	174 (34)	0.0001*
ABI	1.21 (0.2)	1.20 (0.2)	NS
Current smokers, *n* (%)	769 (22)	45 (29)	0.05^†^
Self-reported abnormal total cholesterol, *n* (%)	1352 (37)	77 (45%)	0.001^†^
Self-reported abnormal glucose, *n* (%)	273 (8)	20 (12)	0.04^†^
Pos. family history, *n* (%)	1365 (38)	67 (39)	0.6
METs	7.8 (3)	6.4 (2)	0.0001*
Blood pressure groups			
Group I (reference)	2125 (58%)	44 (26%)	
Group II	719 (20%)	56 (33%)	
Group III	467 (13%)	30 (18%)	
Group IV	214 (6%)	14 (8%)	
Group V	114 (3%)	26 (15%)	0.0001^†^

Data are mean (SD) or *n* and proportion (%), *t-test, ^†^ *χ* ^2^ test.

SBP80: systolic blood pressure at the exercise level of 80 Watts.

SBP150: systolic blood pressure at the exercise level of 150 Watts.

SBPMax: maximum systolic blood pressure during exercise.

ABI: ankle brachial index.

METs: physical working capacity in metabolic equivalents.

Please see Section 2 for the explanation for the blood pressure groups.

**Table 2:** Hazard ratios (HR, 95% Confidence Interval (CI)) of cerebrovascular events according to the specified blood pressure groups (total *n* = 3808, no of events = 170).

Blood pressure group	Model 1			Model 2		
	HR	95% (CI)	*P* values	HR	95% CI	*P* values
Group I	1	(Reference)		1	(Reference)	
Group II	2.69	1.78–4.07	<.0001	2.24	1.43–3.52	<.0001
Group III	2.60	1.60–4.20	<.0001	2.09	1.21–3.61	.008
Group IV	2.71	1.44–5.10	.002	2.37	1.31–4.69	.007
Group V	7.82	4.67–13.12	<.0001	5.78	3.31–10.10	<.0001

Model 1: adjusted for age and sex.

Model 2: adjusted for age, sex, BMI, resting brachial systolic blood pressure, smoking, early parental cardiovascular disease and physical working capacity (METs), self-reported elevated cholesterol, and abnormal blood glucose.

Please see Section 2 for the explanation for the blood pressure groups.

**Table 3:** Hazard ratios (HR, 95% Confidence Interval (CI)) of cerebrovascular events by quartile of pulse pressure.

	Model 1			Model 2		
	HR	95% CI	*P* values	HR	95% CI	*P* values
Quartile I	1	(Reference)		1	(Reference)	
Quartile II	0.91	0.54–1.53	.7	0.92	0.55–1.56	.7
Quartile III	1.28	0.80–2.07	.3	1.21	0.75–1.95	.4
Quartile IV	1.62	1.04–2.65	.03	1.47	0.95–2.33	.08

Model 1: adjusted for age and sex.

Model 2: adjusted for age, sex, BMI, smoking, early parental cardiovascular disease and physical working capacity (METs), self-reported elevated cholesterol, and abnormal blood glucose.
